# Lost in chemical space? Maps to support organometallic catalysis

**DOI:** 10.1186/s13065-015-0104-5

**Published:** 2015-06-18

**Authors:** Natalie Fey

**Affiliations:** School of Chemistry, University of Bristol, Cantock’s Close, Bristol, BS8 1TS UK

**Keywords:** Chemical space, Computational chemistry, Design of experiments, Structure–property relationships, Chemoinformatics, Drug discovery, Organometallic catalysis, Principal component analysis

## Abstract

Descriptors calculated from molecular structures have been used to map different areas of chemical space. A number of applications for such maps can be identified, ranging from the fine-tuning and optimisation of catalytic activity and compound properties to virtual screening of novel compounds, as well as the exhaustive exploration of large areas of chemical space by automated combinatorial building and evaluation. This review focuses on organometallic catalysis, but also touches on other areas where similar approaches have been used, with a view to assessing the extent to which chemical space has been explored.

**Graphical abstract:**
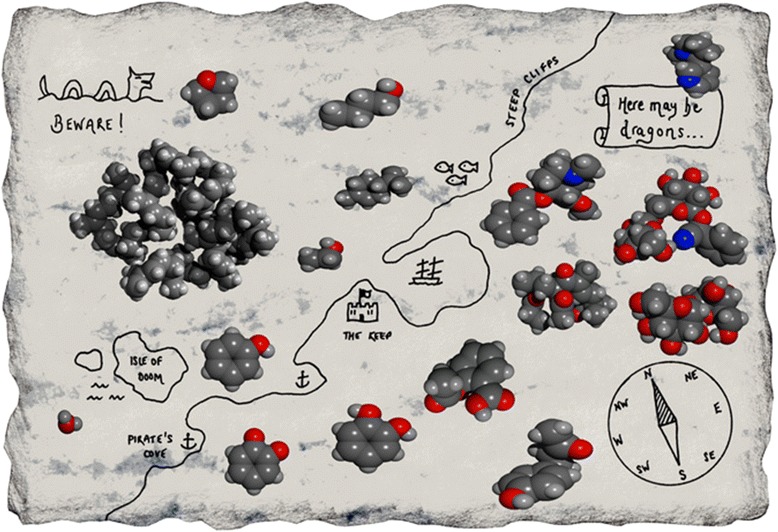
Cartoon representation of a chemical space map.

Cartoon representation of a chemical space map.

## Introduction

Much of modern life relies on maps of familiar and foreign territories, whether they are used to plan a journey, deliver goods to the right address, or to display information about the health and wealth of people. Maps were once a luxury of the ruling classes and often woefully inadequate, but nowadays satellite mapping and the global positioning system (GPS) put a wealth of information in the hands of ordinary citizens at a variety of scales and resolutions, and both *terra incognita* and “there be dragons” have become relics of the past. And while many areas of science are also getting mapped in different ways, ranging from the universe and other planets to the genomes of living creatures and the properties of elements in the periodic table, graphical depictions of the whole universe of chemically-accessible molecules are rare and substantially incomplete.

There is an issue of scale, where, even when limiting it to organic chemical space, usually involving compounds of C, H, N, O, S and the halides, as well as P in some cases, and restricting compound size to drug-like molecules of interest to the pharmaceutical industry, somewhere between 3.4 × 10^9^ [1] and 1 × 10^200^ compounds [2] may need to be considered (1 × 10^60^ is the number given most frequently [3, 4]). Of these, available databases of known compounds capture in the tens of millions of structures [4], revealing a vast discrepancy between what has been synthesised/characterised and the compounds we think could be made. Consideration of the so-called chemical universe, extending beyond organic compounds to encompass all areas of chemistry, lies even further beyond our understanding, reach and data storage capabilities.

The characterisation of unknown chemical compounds relies on calculated property descriptors (the term parameters is commonly used interchangeably, especially in organic and organometallic chemistry) and the computational mapping of chemical space has become increasingly viable with the growth of cheap computing hardware, extensive data storage and networked electronic access. Arguably, the necessary software and computing power are now within reach of many researchers in the chemical sciences, and experiments of the future may be preceded by a computational characterisation of compounds of interest, which, when coupled with predictive models, could lead to the selection and prioritisation of the most promising synthetic routes and products [4, 5].

In a world of increasingly scarce resources and tighter regulations, such an approach holds great promise and this review will seek to provide an overview of recent efforts (predominantly published since 2010) to map different areas of chemical space with calculated descriptors derived from molecular structures. While the primary focus will be on representative examples from organometallic homogeneous catalysis, bridging both catalyst development and their applications to organic synthesis, some forays into other areas of chemical space, especially target substrates and products of catalysis, will also be mentioned, with a view to providing an idea of how much of the chemical universe has been explored computationally to date.

## Review

### Why map chemistry?

In broad terms, calculated property descriptors are processed into maps of chemical space^1^ for three different, sometimes connected, purposes: 1) fine-tuning and optimisation, 2) screening and selection, and 3) exploration. (Adapted from Yang, Beratan et al., ref. [6]).

In the development and improvement of catalytically active complexes, ligands (i.e. ions or small molecules binding to transition metal centres) are a convenient way of fine-tuning catalyst performance once a viable reaction has been optimised to be catalytic. Similarly, the properties of a desirable product (e.g. a compound with potential uses as a pharmaceutical) can be optimised by varying its substituents. These improvements can be guided by computation, allowing researchers to predict the effect of modifications on a compound of interest before its synthesis is carried out. Here both the interpretation of available data on related compounds and the likely mechanism of reaction, often in terms of the relative importance of steric and electronic effects, and the making of predictions for novel structures, may be attempted. In consequence, 3D molecular structures are generally calculated with electronic structure methods^2^ and used to determine relatively sophisticated descriptors specific to the chemistry of interest, such as ligand binding energies in organometallic complexes [7–10] and IR stretching frequencies [8].

The area of *selection* includes automated virtual screening to identify the most promising targets for synthesis (note that it can also be used to identify protein targets in medicinal chemistry, but this lies outside of the scope of this review), but it can also mean evaluating novel designs before their experimental realisation by setting them into a context of known compounds, usually those with desirable properties. Here, fast structure generation can become important for large-scale screening efforts [4], but 3D structures [11], albeit at times calculated cheaply [12],^3^ are still used in smaller databases. In addition, studies are likely to include a figure-of-merit, related to the catalytic cycle [13, 14] or the key property considered to affect properties and activity [15], to assess structure–property/activity relationships more closely. Databases generated are often larger and descriptors can be selected for speed of their evaluation, sacrificing detailed chemical interpretation to some extent.

Finally, where *exploration* is the main target, generation of a large and diverse set of molecular structures (sometimes termed “exhaustive enumeration”) is as important as the fast characterisation of these structures with suitable descriptors [6, 16]. Those which can be calculated from simple structural formulae, i.e. topological and 2D descriptors, are more likely to be used, as they are often relatively cheap to calculate and will not require optimisation and conformational searching of 3D structures.

**Fig. 1:**
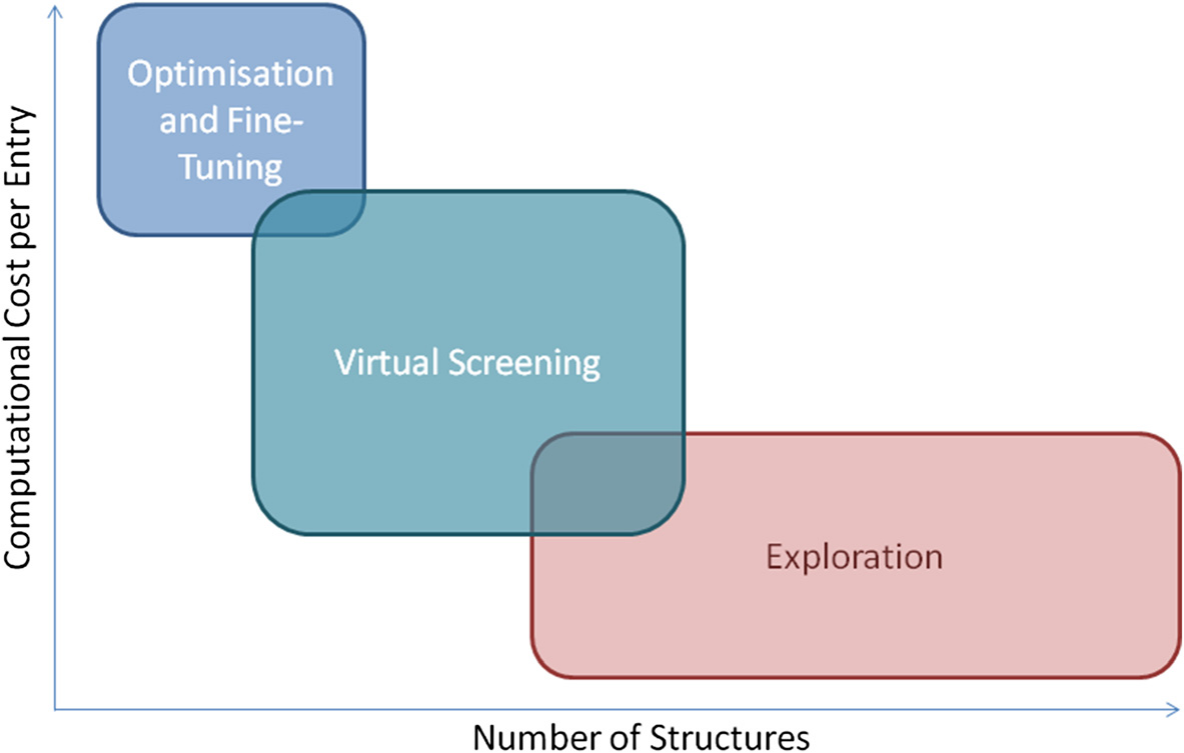
Schematic illustrating the necessary trade-off between computational cost per entry and the number of structures considered in a database of compounds characterised with calculated property descriptors

As indicated above, there is some overlap between these three reasons for mapping chemical space in individual studies, e.g. an exhaustive exploration of chemical space may later be followed by screening subsets of such compounds with calculated figures of merit [16]. At the other end of the spectrum, as datasets developed for optimisation grow in size and sample chemical space better, they can be augmented with suitable calculated figures of merit and then also used for virtual screening [17]. Nevertheless, this classification provides a useful link with the numbers of structures calculated, increasing on going from fine-tuning to exploration (illustrated in Fig. 1). Similarly, this links to the computational cost per entry and the accuracy of the descriptors used, from full quantum chemical structural characterisations to fast calculations of topological descriptors, and, correspondingly, from detailed mapping of structural and electronic properties, retaining close links to the mechanism of reaction, to coarse bins of structural similarities.

### Principal component analysis

In the extreme, only two or three descriptors may be considered to characterise compounds, facilitating the generation of maps from simple plots, such as Tolman’s map of cone angles and electronic parameters [18, 19]. For larger databases with multiple (correlated) descriptors, a range of statistical approaches are available to convert data into maps of chemical space, and of these, principal component analysis (PCA) is used most widely, likely because the approach is implemented in many data analysis packages. It is worth noting here that a range of other approaches have been used, especially in drug discovery, such as self-organising/Kohonen maps (SOM), generative topographic maps (GTM) and a range of clustering approaches, and these have recently been reviewed [20]. While detailed discussions of this approach can be found in a variety of books (e.g. [21, 22], it can be summarised in brief as follows:

PCA is a statistical projection approach which can be used to capture correlated data in fewer, orthogonal dimensions, allowing data similarity to be visualised as distance in low-dimensional plots of the resulting principal components (PCs). These are linear combinations of the original descriptors, with coefficients determined by a constrained optimisation process, designed to capture as much of the variation in the data set as possible in fewer, orthogonal PCs. Nevertheless, the technique generates the same number of derived descriptors as the original dataset used, albeit of decreasing importance, such that the first 3–5 PCs often capture 70–90 % of the variation in the dataset. Coefficients (also called loadings) can be used to determine the importance of individual descriptors to the variation in the dataset and so derive interpretations, but it is worth bearing in mind that PCA is not statistically robust [7] and changes to the dataset can substantially affect the PC composition, hindering their detailed chemical interpretation. Nevertheless, a rough resolution of steric and electronic effects can often be achieved. Each compound in the database is then described by its PC scores, and score plots (scatter plots of the first few PCs) can be used to visualise the data set, with points close in chemical space similar, while increased distance relates to greater differences.

### Focus on fine-tuning and optimisation

Organometallic chemistry has a well-established tradition of using ligand descriptors to identify steric and electronic effects on the properties and behaviours of transition metal complexes [5, 18, 23], because ligands often provide a convenient approach to the fine-tuning and optimisation of complex properties. Similarly, organic chemistry relies on a semi-quantitative understanding of the steric and electronic properties of substituents, rooted to some extent in Hammet and related substituent parameters [24] used to identify linear free energy relationships. Efforts in both fields have also sought to capture chirality and hence make predictions about asymmetric induction and selectivity [25–27]. Structure–property and structure-reactivity relationships can help to interpret experimental observations and, where sufficient data are available, even to make predictions about related compounds for which descriptors have been determined, but which have not been studied experimentally.

**Fig. 2:**
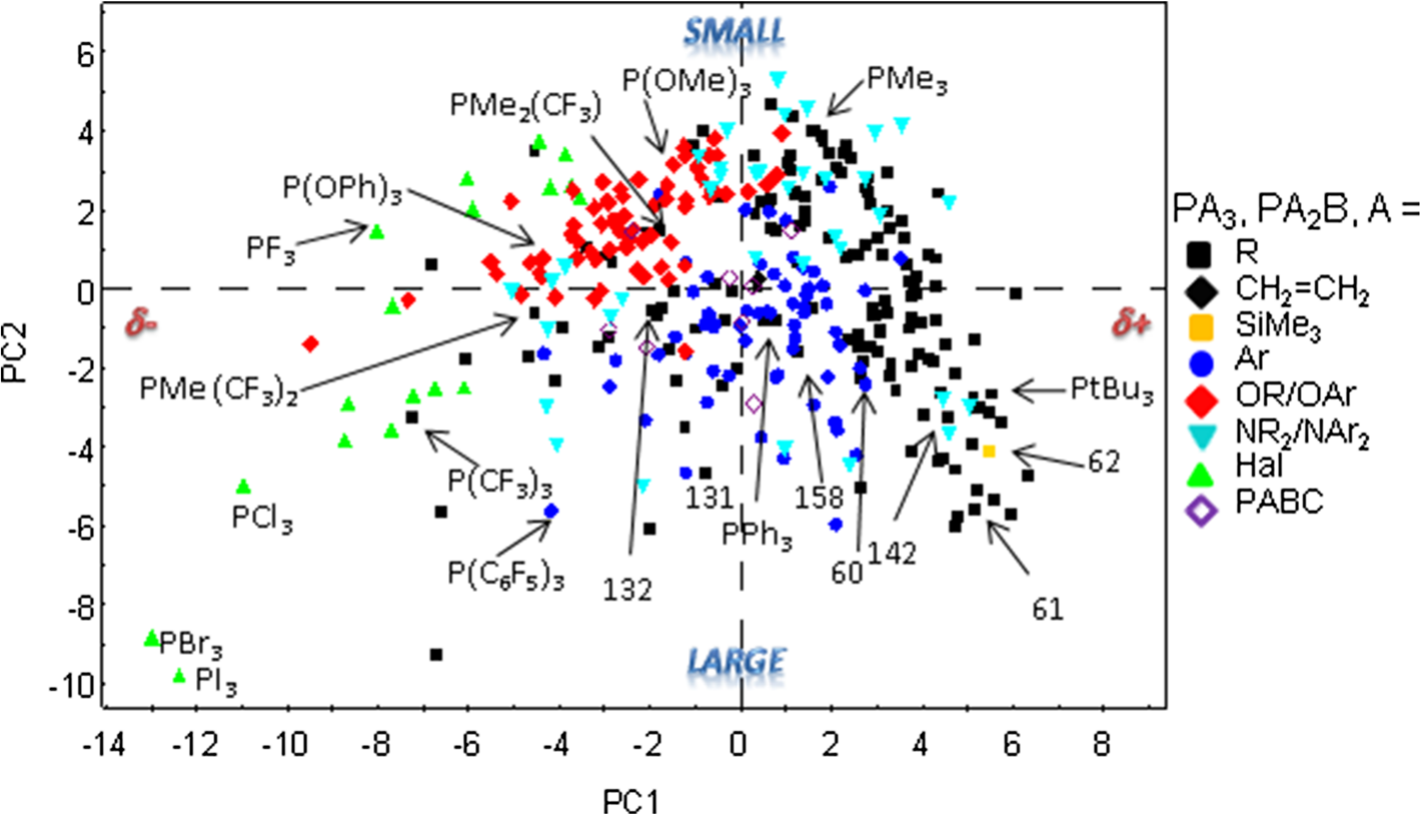
Map of ligand properties generated by principal component analysis of 28 ligand descriptors capturing the structures and energies of 348 P-donor ligands through DFT-calculated data, collected in LKB-P. [28] The principal components are linear combinations of the original descriptors derived to capture most of the variation in fewer uncorrelated descriptors (65 % in this case). Each symbol corresponds to a ligand, and shape and color are determined by substituents. Reprinted with permission from Ref. [28]. Copyright 2010 American Chemical Society

In recent years, calculated parameters have gained increasing acceptance in this area, not in the least because these enable the consideration of novel compounds before their synthesis is attempted. Perhaps the best-characterised class of ligands are monodentate P-donor ligands for which a range of approaches have been described, and these have been reviewed in detail elsewhere [18]. Possibly the biggest database for these ligands has been developed by a consortium of academic research groups at the University of Bristol and data for in excess of 350 ligands have been published to-date [7, 17, 28], with over 1000 held in-house. Other types of ligands have also been characterised computationally, including anionic ligands [29], carbenes [9, 30–32] and other C-donor ligands [33], and bidentate P,P and P,N-donor ligands [34–36]. Some of these datasets have been processed into maps of ligand space, either by plotting key descriptors against each other directly [8, 37], or by processing multiple descriptors with principal component analysis (Fig. 2 shows an example of the LKB-P map [28] of ligand space) [7, 28, 30, 33–36] (for a brief summary of this statistical method, see above). These maps can be used to quantify ligand similarities and set ligands into context [28], allowing evaluation of novel designs to precede synthesis. This has recently been used by researchers in Bristol to predict and then deliver novel fluorophosphine ligands R_2_PF, giving active catalysts for hydroformylation and hydrocyanation, as suggested by their proximity to phosphite ligands on the LKB-P map of ligand space [17] before synthesis.

Ligand descriptors can also be used to analyse a broad range of response data from both experimental and computational studies, allowing their interpretation (and in some cases prediction) in terms of steric and electronic effects [14, 18, 28, 38]. Ligand effects on transition metal complexes are relatively subtle and it can be challenging to separate steric and electronic effects, so these studies generally rely on electronic structure calculations [18] (most commonly using density functional theory (DFT), although some of these studies used semi-empirical [8] and QM/MM approaches [37]) to optimise structures and calculate/extract descriptors. The associated computational cost has limited the size of databases. Ligand structures characterised by different computational descriptors, albeit at detailed resolution, likely number in the low thousands, with little overlap between different ligands,^4^ perhaps best compared to a map of the local area or private garden.

### Focus on selection and screening

**Fig. 3:**
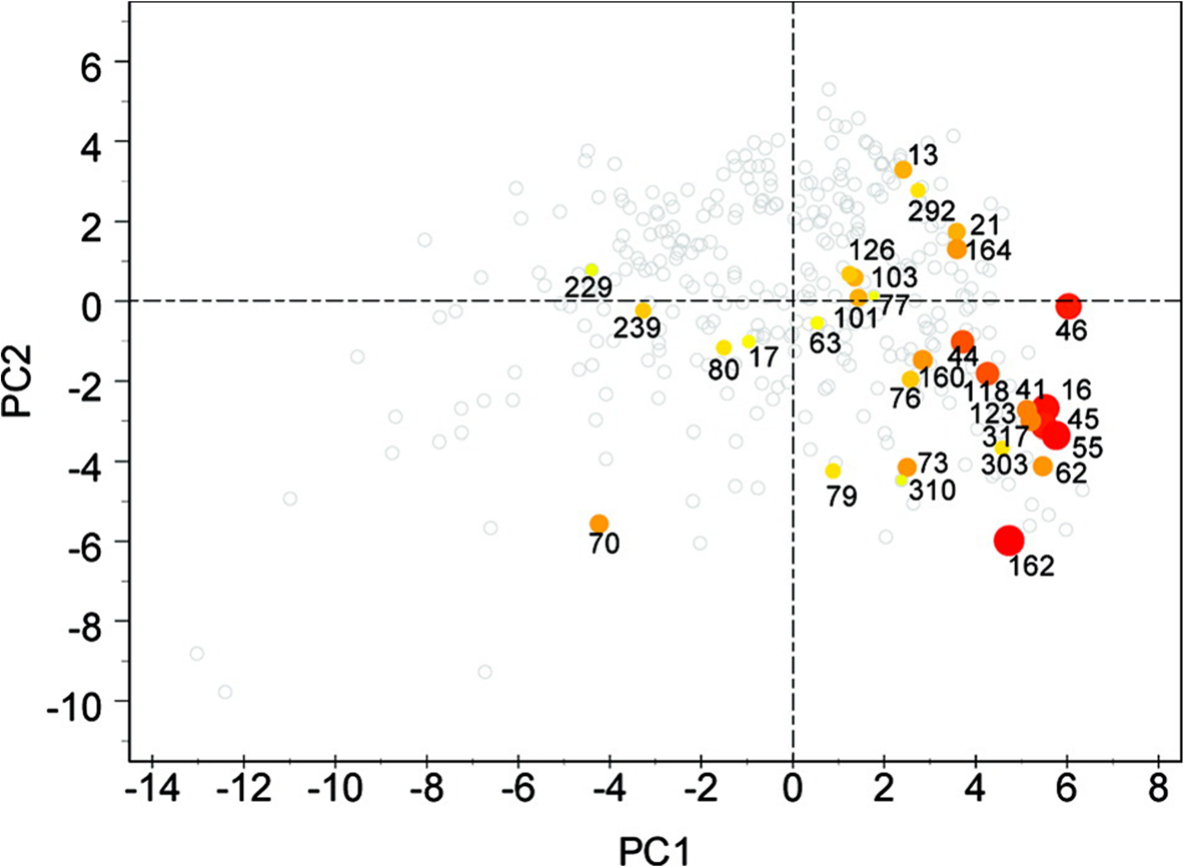
Projection of FRET yields for palladium-catalyzed amination of aryl bromide [56] onto LKB-P map. [28] Spot size and coloring relate to yield, with dark red, large spots corresponding to the highest yields (55, 69 %; 162, 80 %) and small yellow spots corresponding to lowest yields (77, 8 %; 310, 9 %). See original publication for ligand numbering. Reprinted with permission from Ref. [28]. Copyright 2010 American Chemical Society

Some of the ligand property maps generated from calculations with electronic structure methods and surveyed in the preceding section have also been used to support catalyst screening and experimental design (Design of Experiments, DoE). This can take the form of simply projecting a desirable property or response onto a map of ligand space, as demonstrated with LKB-P (Fig. 3) [28, 39] and, if a cluster of ligands is found to exhibit this property, testing structures in the same area experimentally.

**Scheme 1:**
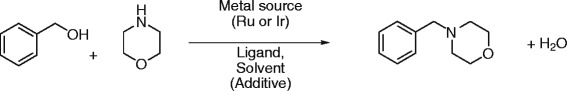
Borrowing hydrogen model reaction optimised as described in reference [39]

Where sufficient data for training regression models are available, quantitative prediction can also be attempted [12, 14, 28, 40]. Maps can be used to select ligands for experimental screening, and their use with Design of Experiment (DoE) approaches has recently been highlighted by Moseley and Murray (M&M) [39], presenting a case study of reaction optimisation for ruthenium and iridium catalysis of borrowing hydrogen reactions (Scheme 1). In experimental design, compound data is discrete, rather than continuous [39], but its use is possible, and M&M describe the use of a cube on 3D maps of ligand space generated from PCs1–3 to select compounds for screening.

However, databases developed primarily for screening often use lower levels of theory to calculate descriptors, although separation of steric and electronic effects is still feasible, and the automated building of structures plays a more important role. These issues have been explored extensively by the group of Rothenberg based in Amsterdam [15, 41] and the group have reported a number of studies: Ligand and solvent effects have been screened, [42, 43], using response data harvested from the literature and combining it with a range of fast-to-calculate 2D and 3D descriptors. In addition, the bite angles and flexibility of bidentate P,P donor ligands have been investigated by Rothenberg’s group, using topological descriptors, with a view to demonstrating that cheap calculation approaches can give useful models for prediction and so be applied to *in silico* screening of large ligand and catalyst libraries [15]. A broader range of descriptors was later used by the same group to evaluate a library of ligands with a view to maximising their diversity, mapping ligand diversity in 2D- and 3D space [12]. This approach was also used to generate and evaluate a catalyst library from fragments [40], a proof-of-concept study designed to identify novel catalysts for Rh(I)-catalysed hydroformylation reactions. Unfortunately, the latter study provides little detail on the descriptors used, nor indeed the novel ligand designs identified.

**Scheme 2:**
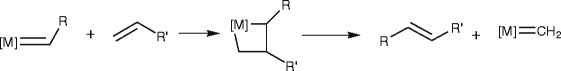
Alkene metathesis

For organometallic catalysis, and alkene metathesis (Scheme 2) catalysed by ruthenium carbene complexes in particular, the group of Jensen based in Bergen currently leads in the automation of structure generation; starting with ligand screening by a range of descriptors available in chemoinformatics software [14], they have since described the automated assembly and screening of a broad range of ligands [13], as well as developing their own modifications and rules to make approaches from other areas of chemistry more suitable for organometallic catalysis [11, 44]. These studies have adapted combinatorial virtual synthesis algorithms for use with transition metal centres, and combine this with a range of descriptors and response data to pursue the discovery of viable novel catalysts; they are not usually processed into formal maps of chemical space, though.

Moving beyond the evaluation of organometallic complexes, the virtual screening of drug-like molecules, either based on their structures (evaluating similarity to known drugs, bioavailability, diversity etc.), or based on their interaction with known protein targets, is perhaps the most substantial field using calculated descriptors in screening. Indeed most of the examples in organometallic catalysis have used and adapted tools from this area [13, 43]. While such molecules may be of interest to organometallic chemists as synthetic targets and application examples for novel catalysts, an exploration of this area lies outside the scope of the present review. Note that it has been reviewed extensively, see e.g. refs [20, 45–49]. In this area, the need for greater diversity in terms of the structural scaffolds used is a persistent theme [50–52] and novel catalytic routes may open up greater diversity in the future.

Studies aimed at selection and screening thus use a broad range of descriptors. However, in very general terms, large libraries are usually associated with descriptors that are cheap to calculate from basic connectivity information (topological and 2D), while smaller screening studies are more likely to use quantum chemistry to determine electronic properties from 3D structure, often linked quite closely to the likely mechanism of reaction; it is perhaps worth noting at this stage that the groups in Bristol, Bergen and Amsterdam have all reported on their efforts to map the chemical space relevant to organometallic catalysis over long time periods, thus accommodating the computational efforts necessary. Similarly, combinatorial building approaches are most likely to be used where large and diverse databases are of interest, whereas smaller-scale screening can be accomplished without such automation. In addition, many of these studies reach beyond structural similarity and often use a figure-of-merit which is related to the descriptor data by regression models to make predictions for novel designs. While for organometallic compounds database size remains in the thousands, potentially extending to tens of thousands, pharmaceutical virtual screening routinely accesses larger databases and even databases of known compounds capture around 30 million structures [4] in this area, necessitating compromises to reduce the computational costs of descriptor calculations. Screening maps thus vary substantially in scale and resolution, and might be likened in range to maps of cities and counties at the lower end, exploring entire continents in other cases.

### Focus on exploration

Analysis of known successful drug molecules by structural descriptors has highlighted that structural diversity in this group of compounds is relatively limited (described as “chemical clichés” [4]), presumably constrained by known synthetic routes as well as what might be termed “cultural preferences” for familiar reagents and reactions. In both organometallic catalysis and organic, drug-like chemical space, several attempts have been made to increase diversity by combining automated molecular assembly with structural descriptors. Again, there is overlap here with the virtual screening described above, e.g. Rothenberg’s work on bidentate ligands [12], but figures of merit are generally not evaluated leaving structural similarity to known compounds as the main criterion.

**Fig. 4:**
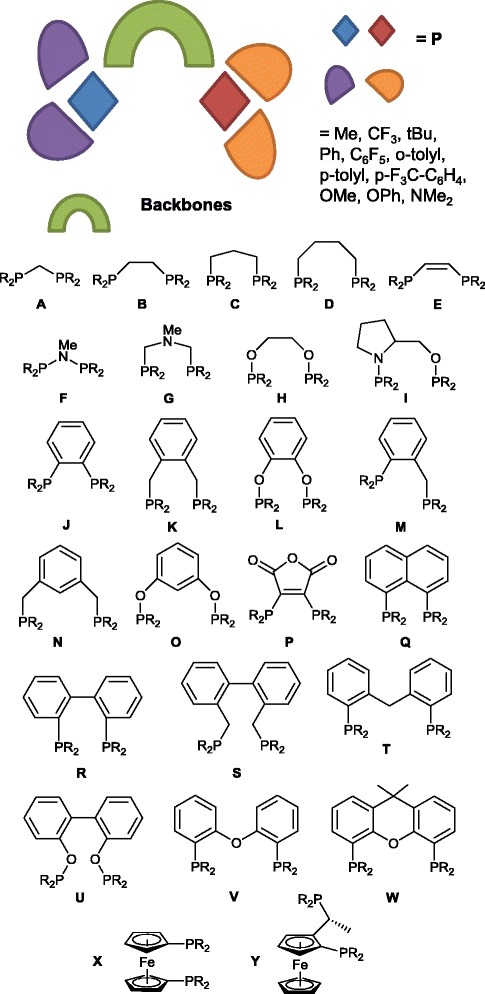
Donors, substituents and backbones sampled in LKB-PP_screen_. Adapted from reference [35] by permission of the Royal Society of Chemistry and reproduced from reference [5] with permission from WILEY-VCH Verlag GmbH & Co. KGaA, Weinheim

**Fig. 5:**
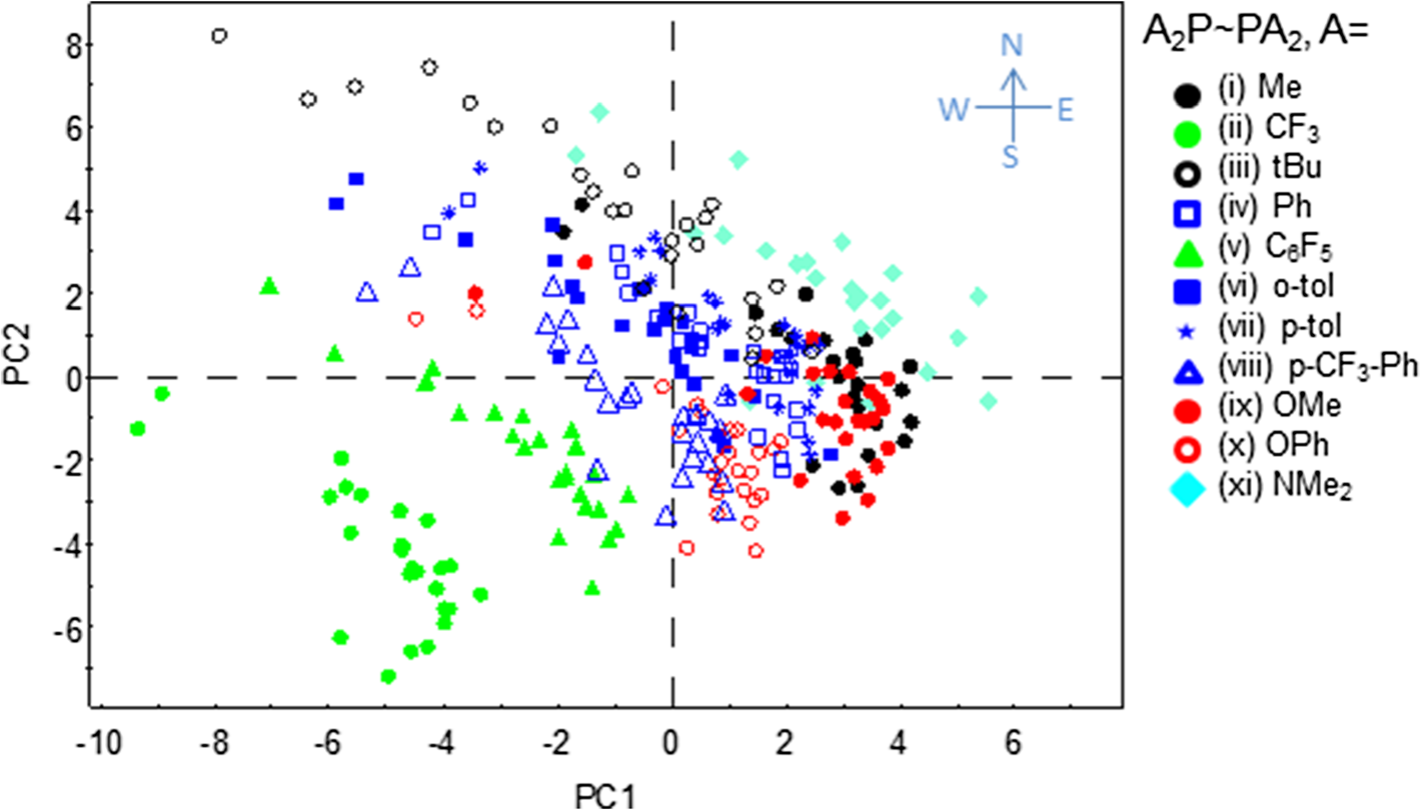
Principal component score plot showing the first two principal components (PC1 and PC2) generated by analysis of the full LKB-PPscreen database of 28 steric and electronic descriptorss, calculated for 275 ligands (see [35] for details). Each symbol corresponds to one ligand, with colour/shape representing different substituents, and the first two PCs capture ca. 56 % of variation in data. Reproduced from ref. [35] with permission from The Royal Society of Chemistry

Analysis of bidentate ligand space as mapped by Bristol’s LKB-PP [34, 36] indicated relatively poor sampling of ligand space by ligands used experimentally, and a more detailed, exploratory scan of this area of chemical space was performed by combining known backbones with a broader range of substituents (most experimental studies have focussed on backbones and simply used Ph substituents) to give 275 ligand structures (Fig. 4) [35]. These were then evaluated with the LKB descriptors developed previously [34, 36], using DFT calculations, but calculations have been simplified and automated more substantially to streamline the evaluation of these ligands. Data analysis (Fig. 5) suggested that both backbones and substituents lead to ligand property variations and that new areas of bidentate P,P donor ligand space could be accessed by introducing greater variability in the substituents used experimentally. It is worth noting that this could be expanded further to consider larger numbers of backbones and substituents, this work mainly served as a proof-of-concept.

Mapping the properties of molecules based on their structures is a key feature of research and prediction in the development of potential drug molecules, and here the largest numbers of compounds have been characterised computationally. Two examples are included here to give an overview of the mapping of substrates and reaction products potentially accessible to organometallic catalysis.

The group of Reymond based in Berne aims to map drug-like chemical space and then mine it for possible new structures (“The Chemical Space Project”, http://www.gdb.unibe.ch/). To date, their largest database, generated by exhaustive enumeration of chemically feasible bonds between C, N, O, S and the halogens contains compounds with up to 17 of these heavy atoms (GDB-17, 1.7 × 10^11^ molecules) [53]. These structures have been characterised with 42 molecular quantum numbers (MQN), topological descriptors not requiring the optimisation of structures, but allowing coarse allocation to bins of similar compounds. Although so-called polarity counts are included, many of these descriptors focus on molecular size. The group also reports various screening and selection projects based on their GDB databases, exploring fragrances [54] and searching for potential replacements for known pharmaceuticals [4, 16, 55]. These analyses use smaller subsets of the databases, and rely on simplified figures of merit to predict likely performance based on the MQNs.

In contrast, the groups of Yang and Beratan argue that the exhaustive enumeration of possible structures is not necessary [6], describing instead a genetic algorithm designed to maximise structural diversity without building all possible compounds. This allows them to select a representative and maximally diverse subset of the small molecule universe (SMU), which captures similar diversity to Reymond’s approach in fewer steps. As restrictions on molecular size can be relaxed, this approach allows the exploration of more of chemical space (3.6 × 10^9^ structures). They use different topological descriptors, again easy to calculate, include an evaluation of synthetic accessibility and favour self-organising maps (SOM) for data visualisation. They have also used the resulting dataset to search for drug-like molecules, but this is not described in detail.

These studies show that the computational exploration of unknown chemical space is feasible, and new maps continue to be drawn at a variety of scales, even extending to a coarse mapping of the small molecule universe, akin to maps of the world and the visible night sky.

## Conclusions

The studies summarised here show that subsets of chemical space have been mapped with calculated descriptors, ranging in sophistication from topological descriptors derived from structural formulae to descriptors specific to organometallic catalysis, extracted from quantum chemical calculations. At all levels, these descriptors can, at least coarsely, allow to distinguish and quantify the contributions of steric and electronic effects to compound properties. Where suitable response data are available, regression models can also be derived, allowing interpretation and at times predictions to be made. However, models and maps will only ever be as good as the data used to generate them, and even predictions based on quantum chemical descriptors able to distinguish very subtle modifications to the electronic structure of catalysts will fail if the mechanism of reaction changes, so these need to be tensioned against experimental data at every opportunity.

Arguably, any calculation of structural descriptors contributes to the mapping and exploration of chemical space, but many studies also resort to statistical approaches to visualise results, and here principal component analysis is perhaps most widely used. On the resulting maps of chemical space, proximity points to greater similarity, and such maps, as well as the underpinning descriptors, have been used for the optimisation, screening and exploration of compounds with different levels of resolution. It is worth bearing in mind that PCA is not statistically robust, so maps will change as the compound database evolves, and that descriptors and analyses can sacrifice some of the links with chemical behaviour to enable, for example, a larger database to be generated. Again, tensioning analyses against chemical insights and experimental data is invaluable in demonstrating the utility of large-scale mapping of chemical space.

In the end, the map analogy is important here–just as world maps do not have the resolution necessary to show trees and houses, large-scale exploratory mapping of the chemical universe is not going to translate into sophisticated predictive models for all compounds captured, and the intended application very much determines the computational approach used. Compound descriptor data are gaining importance in different areas of chemistry, and are likely to play a key role in progressing computational prediction to increasingly precede chemical synthesis, but these approaches are strengthened by close links to experimental reality.

## Abbreviations

DFT: Density functional theory
DoE: Design of experiments
GDB: Global database
GPS: Global positioning system
IR: Infra-red
LKB: Ligand knowledge base
MM: Molecular mechanics
MQN: Molecular quantum numbers
NMR: Nuclear magnetic resonance
PCA: Principal component analysis
PC: Principal component
QM: Quantum mechanics
SMU: Small molecule universe

## Footnotes

1 Arguably, any property descriptor contributes to our understanding of the chemical universe and here, while discussion will focus on those that have been processed into 2D and 3D maps, related studies will also be mentioned.

2 These encompass theoretical approaches focussing on the electronic structure of compounds, i.e. semi-empirical, Hartree-Fock (HF), density functional theory (DFT) and post-HF approaches. They are generally more demanding of computational resources than classical force-field approaches (molecular mechanics/MM); the terms quantum mechanics (QM) or quantum chemistry are also used in this context.

3 The cost of a calculation arises from the processing power and storage capacity needed, as well as the time taken to produce the desired output. Electronic structure methods (see previous note) are generally more expensive than molecular mechanics (MM), where, with appropriate parameterisation, reasonable structures can often be obtained quickly.

4 Metal-ligand binding changes substantially for different ligand classes, and these differences are difficult to accommodate with a single set of descriptors. Even where this has been attempted, responsiveness of descriptors may vary and PCA can pick up on these differences, showing clustering of similar ligands with limited overlap.
